# Hell’s Itch: A Case Series of a Debilitating Post-Sunburn Pruritic Syndrome in a Healthy Young Adult

**DOI:** 10.3390/reports8040217

**Published:** 2025-10-28

**Authors:** Precious Ochuwa Imokhai, Alexandra DeVries, Katelin Ball, Brandon Muse, Benjamin Brooks

**Affiliations:** 1 Montana College of Osteopathic Medicine, Rocky Vista University, Billings, MT 59106, USA; 2 College of Medicine, Rocky Vista University, Parker, CO 80134, USA; 3 Department of Research and Scholarly Activity, Rocky Vista University, Ivins, UT 84738, USA

**Keywords:** Hell’s Itch, post-sunburn pruritus, Β-alanine, TRPV1-mediated itch, neurogenic inflammation

## Abstract

**Background and Clinical Significance**: Hell’s Itch is a rare, intensely uncomfortable post-sunburn condition with burning pruritus emerging 24–72 h after UV exposure. This condition often goes unrecognized and is frequently misdiagnosed by healthcare providers due to a lack of knowledge and familiarity. Standard antipruritic measures are often ineffective, and patients frequently rely on anecdotal self-management. **Case Presentation**: Three healthy adult males between 23 and 28 years old experienced multiple episodes of delayed-onset intense pruritus following moderate-to-severe sun exposure. The patients experienced a burning or stinging pain which they described as “fire ants” or “thumbtacks,“ and their symptoms started between 24 and 72 h after sun exposure without any rash or fever symptoms. The patients did not achieve symptom relief from standard treatments which included oral antihistamines and topical lidocaine, NSAIDs, aloe vera, and cold compresses. The patients received β-alanine treatment through pre-workout supplements or pure powder after consulting non-clinical sources. Each patient ingested β-alanine and reported rapid relief (itch 8–10/10 → 1–2/10) lasting 2–3 h. The only adverse effect reported by one patient was mild paresthesia. **Conclusions**: This case introduces β-alanine as a potential off-label therapy for Hell’s Itch and emphasizes the psychological burden and clinical complexity of the condition. While anecdotal, further study is needed to elucidate the mechanism of action of β-alanine in relieving symptoms of Hell’s Itch, as well as assess safety and efficacy in controlled settings. Increased clinical awareness of Hell’s Itch may reduce patient distress and improve management strategies.

## 1. Introduction and Clinical Significance

Sunburn represents a typical dermatologic condition that resolves on its own, but Hell’s Itch emerges as a severe and poorly recognized condition among patients. The condition appears 24–72 h after moderate-to-severe sun exposure when patients develop an unbearable burning sensation that they describe as intense deep pruritus. The condition stands out because of its extreme severity and unresponsiveness to standard antipruritic treatments and its intense symptoms that exceed the visible skin damage. The medical community has not included Hell’s Itch in dermatology textbooks nor has it received sufficient attention in the clinical literature despite frequent patient reports in online forums.

The exact mechanisms that drive this condition remain unclear to medical professionals. Experimental research indicates broadband UVB exposure activates TRPV1-positive dorsal root ganglion neurons to produce intense itch sensations that occur independently of mast cell degranulation and histamine signaling [1]. This may explain the limited benefit of antihistamines in many patients [2]. The pruritic response seems to result from neuroimmune sensitization together with non-histaminergic pathways that involve TRPV1 ion channels and inflammatory cytokines [1,2,3]. The current medical literature shows that topical anesthetics and cool compresses provide temporary symptom relief, but there is no established effective treatment for this condition [4].

The case series describes three healthy young adult males who experienced multiple Hell’s Itch episodes, including episodes treated with oral β-alanine in its different forms. The documented case represents one of the first instances where β-alanine provided fast relief from Hell’s Itch symptoms to patients who normally use this supplement for sports performance. This study adds to the scarce scientific knowledge about Hell’s Itch and suggests a potential therapeutic approach that could guide upcoming clinical investigations.

Clinical Significance: This case highlights Hell’s Itch as a debilitating, underrecognized condition that may be mediated through non-histaminergic neuroimmune pathways. It also identifies β-alanine—a widely available, well-tolerated supplement—as a potential rapid-acting treatment for symptom relief. Given the lack of effective therapies and the psychological burden of this condition, this report emphasizes the need for increased clinical awareness and further research into alternative pruritus management strategies.

## 2. Case Presentation

Cases were assembled from patient self-reports obtained with informed consent. The Rocky Vista University Institutional Review Board granted exemption (Protocol #2025-163; approved 27 June 2025). No protected health information (PHI) was used. This descriptive case series highlights clinical patterns and patient-reported outcomes in a rare post-sunburn pruritic syndrome. Patient-reported itch intensity (0–10 numeric rating scale) was self-estimated retrospectively within 24–48 h of each episode based on the peak itch severity experienced. Patients were instructed to quantify itch intensity as they remembered it at its worst point, consistent with common clinical pruritus severity scales.

### 2.1. Case 1

#### 2.1.1. Initial Presentation

A 26-year-old fair-skinned male with no chronic dermatologic disease, allergies, or systemic conditions developed severe burning pruritus 48–72 h after moderate sun exposure. He reported sensitive skin and mild childhood dryness but no history of atopic dermatitis, allergic reactions, or abnormal responses to sunlight. He was not taking any regular medications and had no significant past medical or surgical history.

#### 2.1.2. Observations

The patient has no chronic skin conditions and no history of atopic dermatitis or allergic reactions. He described having sensitive skin and mild childhood dryness, but no prior unusual reactions to sun exposure. He denied blisters, rash, fever, or systemic signs beyond nausea and tremors. The itching did not respond to cold showers or aloe vera (which exacerbated symptoms), and scratching provided no relief. Lidocaine without aloe and cold compresses offered temporary reprieve. During his most recent episode, oral diphenhydramine and acetaminophen were largely ineffective. The patient has never required hospitalization and has never been clinically diagnosed despite seeking advice from multiple providers.

#### 2.1.3. Diagnosis

The patient self-diagnosed Hell’s Itch through the combination of delayed intense non-allergic pruritus after UV exposure and the absence of rash and previous reports that matched their symptoms. The possible diagnoses included sunburn-associated pruritus, polymorphic light eruption, contact dermatitis, and photo-induced neuropathic itch. The cyclical nature of the condition, the severe burning sensation, and the response to β-alanine made Hell’s Itch the most likely diagnosis.

#### 2.1.4. Treatment

During the patient’s initial episode of Hell’s Itch, he trialed several over-the-counter and supportive therapies with minimal effectiveness. Oral diphenhydramine, acetaminophen, and ibuprofen were taken but did not reduce symptoms. Topical aloe vera and a warm shower exacerbated the itching. Topical lidocaine cream without aloe vera resulted in partial symptom relief approximately ninety minutes after application. Cold therapy, specifically the application of ice cubes directly to the skin, provided short-lived relief and helped prevent scratching, though the underlying pruritus remained unchanged.

During the second episode, the patient again attempted topical lidocaine, but the formulation contained aloe vera and led to a worsening of symptoms. Oral diphenhydramine was also taken without improvement. Based on the patient’s online research, specifically a Reddit thread detailing a possible source of Hell’s Itch symptom relief, he decided to try β-alanine as an off-label treatment. He ingested 3200–4800 mg of a commercially available pre-workout supplement (Six Star Pre-workout Explosion), delivering an estimated 3200 to 4800 mg of β-alanine (1600 mg per serving). Within five minutes, the patient reported substantial relief, with a reduction in itch intensity from 8 to 10 out of 10 down to 2 out of 10. The symptom relief lasted two to three hours, after which he ingested an additional dose. Compared to lidocaine, the patient reported that β-alanine provided faster and more complete symptom control. The patient ingested a pre-workout supplement containing β-alanine along with other active ingredients, including caffeine, niacin, taurine, and betaine. Because these formulations vary by brand and composition, the specific contribution of β-alanine to symptom relief cannot be determined.

#### 2.1.5. Outcome

The patient’s symptoms improved substantially within five minutes of β-alanine ingestion and resolved completely within one hour. No medical care was required during either episode. Although he fully recovered physically, the experience with Hell’s Itch led to long-term psychological effects. He reported persistent anxiety and paranoia about developing future episodes and has limited his time outdoors due to ongoing concern about recurrence. These behavioral changes have affected his quality of life and reflect the psychological burden associated with this condition.

### 2.2. Case 2

#### 2.2.1. Initial Presentation and Medical History

A 28-year-old fair-skinned male of Irish descent with no chronic illnesses, dermatologic conditions, or medications developed intense delayed-onset itching approximately 48 h after moderate sun exposure. He reported no personal or family history of eczema, photosensitivity, or autoimmune disorders. He denied the use of new skincare products or medications and described normal overall health. His lifestyle included frequent outdoor recreation such as hiking and golf.

During the last eight years, he has experienced multiple episodes of intense post-sunburn pruritus which he describes as Hell’s Itch and has occurred six times in total during the early part of summer. The onset of severe pruritus begins 48 h after sun exposure during these episodes. He describes the sensation as “fire ants under the skin” and described it as “being poked with thumbtacks” while his itch reached its peak at 10/10. The itching occurs specifically in sunburned regions but spreads to other areas, continues after scratching attempts, and appears in recurring waves. The patient experienced worsening symptoms when he tried aloe vera gel combined with topical lidocaine and oral ibuprofen. Ice packs and cold showers were ineffective. Hot showers brought him temporary relief from his symptoms. He learned to prevent future episodes by steering clear of known triggers and taking diphenhydramine orally along with ibuprofen and hot showers which provided some relief. He diagnosed himself through Reddit discussions about similar symptoms without visiting a doctor. Episodes caused sleep disruption, anxiety, and functional impairment. He denied suicidal thoughts but expressed desperation and recurring thoughts about skin removal.

#### 2.2.2. Observations

During his most recent episode in June 2025, the patient experimented with oral β-alanine after learning about it on Reddit. He ingested one scoop (1000 mg) of a non-stimulant pre-workout powder (RAW brand) and reported significant symptom relief within 30 min. His itch intensity dropped from 9–10/10 to 1/10 and remained suppressed for approximately 2–3 h. As symptoms gradually returned, he repeated the dose after six hours and again experienced temporary relief.

The patient did not report paresthesia or other adverse effects typically associated with β-alanine supplementation [5]. He did not seek medical consultation, nor was a formal diagnosis established. Despite the subjective relief, the paradox between β-alanine’s known pruritogenic properties and the patient’s response underscores the need for mechanistic clarification [4,6]. His experience reflects a pattern of frustration, experimentation, and reliance on non-clinical sources for managing a poorly recognized condition.

#### 2.2.3. Treatment

During his initial episodes of Hell’s Itch, the patient attempted several conventional sunburn treatments without success. Aloe vera, topical lidocaine, and oral ibuprofen all exacerbated symptoms. Cold therapies, including ice packs and cold showers, were similarly ineffective. He later discovered that extremely hot showers provided short-term relief and became his preferred acute management strategy. In subsequent episodes, he avoided aloe vera and cold applications and instead used oral ibuprofen, diphenhydramine, and hot showers with more consistent—though still limited—symptom relief.

Following a recent episode, the patient trialed β-alanine after reading about its potential effects on an online Reddit forum. He ingested 1000 mg of a non-stimulant pre-workout powder (RAW brand) containing approximately 1000 mg of β-alanine. Within 30 min, he experienced a marked reduction in symptoms, reporting a drop in itch intensity from 9 or 10 out of 10 to 1 out of 10. Symptom relief lasted two to three hours before gradually returning. A second dose was taken six hours later when symptoms re-emerged, resulting in additional temporary relief. The pre-workout powder used by the patient contained β-alanine and other common pre-workout compounds (e.g., taurine, betaine, and L-citrulline). Variability in composition among commercial formulations introduces potential confounding effects, and symptom relief cannot be attributed solely to β-alanine.

#### 2.2.4. Outcome

Β-alanine provided significant short-term symptom relief within 30 min of ingestion during the most recent episode. The patient avoided seeking medical care during all episodes but described the itching as severely distressing and disruptive to his daily life. He reported an inability to work, sleep disturbances, and preoccupation with finding relief during active episodes. Since his first experience with Hell’s Itch, he has become more vigilant about sun protection and UV avoidance but reports on anxiety due to the unpredictability of the episodes.

### 2.3. Case 3

#### 2.3.1. Initial Presentation and Medical History

A 23-year-old fair-skinned male with a history of epilepsy treated with extended-release valproate (1000 mg daily) developed severe pruritus 24 h after two hours of unprotected sun exposure during a 95 °F (35 °C) day. He reported fair, sensitive skin and environmental allergies to pollen but no previous dermatologic diseases. He denied the use of other medications, recent illness, or changes in hygiene or personal care products.

#### 2.3.2. Observations

The pruritus started after a lukewarm shower about 24 h after sun exposure. The patient described the sensation as intense, constant, and non-painful, likening it to “fire ants crawling under the skin”. The itching spread past the sunburned skin areas to primarily affect the lower back and chest but avoided the most red areas on the shoulders. The patient rated the severity at its peak as 10 out of 10. The affected areas showed no rash, wheals, or blistering, but the patient experienced chills, dizziness, and psychological distress. The patient did not have a fever, and scratching did not help.

#### 2.3.3. Treatment

During both episodes of Hell’s Itch, the patient trialed multiple over-the-counter treatments with limited success. In the first episode, the symptoms were triggered following a lukewarm shower and persisted for approximately five hours. He applied an over-the-counter anti-itch cream to his back, which initially provided mild relief. However, when used again during the second episode, it worsened the itching. Oral ibuprofen (four tablets) was taken during the first episode with minimal effect, and diphenhydramine was later tried without significant improvement. A hot shower was the only consistently effective measure for acute relief. In contrast, cold showers and towel drying aggravated the symptoms.

During the second episode, which occurred later the same day, the patient again experienced severe pruritus lasting approximately three hours. Based on information found on Reddit, he decided to trial β-alanine as an off-label treatment. He ingested half a scoop of a powdered pre-workout supplement (RYSE brand, exact dose unknown). Within five minutes, the patient noted a tingling sensation, initially resembling worsening itch, followed by a marked reduction in symptoms. He rated his itch severity as decreasing from 10 out of 10 to 5 out of 10 within ten minutes. No further episodes occurred after this single dose. The RYSE pre-workout supplement ingested by the patient included β-alanine along with additional ingredients typical of commercial pre-workouts, such as caffeine and amino acid derivatives. Because ingredient concentrations were not standardized, β-alanine’s specific role in symptom improvement remains uncertain.

#### 2.3.4. Outcome

The patient experienced substantial symptom improvement within minutes of ingesting β-alanine, with near-complete resolution occurring within the first hour. Although he did not seek medical attention during either episode, he described the pruritus as one of the most physically distressing experiences of his life, second only to epileptic seizures. He reported associated dizziness, chills, and psychological distress, including panic and inability to perform daily tasks. While no self-harming thoughts were endorsed, he expressed empathy toward those who experience such feelings during episodes of Hell’s Itch. Since recovering, he has developed fear around showering post-sunburn and increased anxiety about potential recurrence. These behavioral changes have contributed to a lasting psychological burden and have influenced his willingness to engage in outdoor activities.

Table 1 summarizes the patients’ demographics, triggers, and β-alanine-associated outcomes for quick comparison across cases.

## 3. Discussion

The post-sunburn condition, Hell’s Itch, remains poorly understood and underreported in medical literature. The delayed onset of symptoms between 24 and 72 h and neuropathic characteristics of Hell’s Itch distinguish it from standard sunburn pruritus. Proposed mechanisms include non-histaminergic neurogenic inflammation with TRP channel sensitization and cytokine signaling.

Two patients reported using commercial pre-workout powders, which raises an important consideration of whether other active ingredients beyond β-alanine could have influenced their symptom relief. Along with 1000 mg of β-alanine, one scoop of RAW Pump Non-Stim Pre-workout contains L-citrulline (2.5 g), betaine anhydrous (2.5 g), L-tyrosine (1 g), taurine (750 mg), agmatine sulfate (750 mg), inositol-stabilized arginine silicate (750 mg), and lion’s mane extract (150 mg), as well as chloride, sodium, and potassium. One scoop of Six Star Pre-workout Explosion 2.0, on the other hand, contains 1600 mg of β-alanine plus vitamin C (90 mg), niacin (16 mg), choline (60 mg), L-citrulline (1500 mg), taurine (470 mg), betaine anhydrous (1250 mg), sodium citrate (595 mg), coconut water (100 mg), magnesium oxide (86 mg), sodium chloride (80 mg), dipotassium phosphate (45 mg), L-tyrosine (250 mg), caffeine (160 mg), and choline bitartrate (150 mg), as well as magnesium (50 mg), sodium, and potassium. It is important to consider ingredients like caffeine and niacin as potential modulators of itch perception. Notably, only the Six Star formulation contained caffeine and niacin. While we hypothesize a temporal association between β-alanine and relief of Hell’s Itch due to its known effect on sensory neurons, the potential contribution of these additional ingredients cannot be excluded.

The patient’s reaction to β-alanine suggests a neuromodulatory process, which deserves additional research. This paradox may resemble counter-stimulation therapies such as topical capsaicin, which transiently activate TRPV1-expressing sensory neurons but subsequently suppress itch through desensitization. Hell’s Itch is a debilitating condition characterized by intense, often unbearable pruritus following a sunburn. Despite its severity, clinical awareness remains low, and many patients report unfamiliarity amongst healthcare providers, leading to self-management.

The exact pathophysiology of Hell’s Itch is unclear, but proposed mechanisms include histaminergic involvement and neurogenic inflammation. After UV exposure in the setting of sunburn, histamine is thought to play a role in neurogenic inflammation and the conversion of epidermal keratinocytes into pain-signaling cells [5]. While this is a proposed hypothesis of the pathophysiology, standard antihistamines are generally ineffective, as seen in this case, further emphasizing the need for continued research.

Products containing β-alanine, a non-essential amino acid commonly found in pre-workout supplements, were associated with rapid symptomatic relief of Hell’s Itch in these cases, particularly compared with topical lidocaine. Although its mechanism of action in this context remains unclear, β-alanine is known to activate sensory neuron receptors involved in pain and pruritus, suggesting a possible neuromodulatory effect [7]. This observed relief may reflect a temporal association rather than definitive efficacy, and alternative explanations, including placebo effects or the distracting paresthesia induced by β-alanine, cannot be excluded. Currently, no clinical trials have evaluated β-alanine for Hell’s Itch, and its efficacy remains anecdotal.

These cases highlight not only the underrecognized burden of Hell’s Itch but also the gap in clinical recognition and treatment guidance for those affected. Given the intensity of symptoms and the psychological distress it can cause, there is a need for continued research. Increased clinical awareness, including Hell’s Itch in dermatological primary care, and emergency/urgent care training, and the validation of patients’ symptoms are first steps. Future research should focus on explaining the underlying mechanisms and exploring emerging therapies such as β-alanine through controlled clinical trials. Increasing awareness of Hell’s Itch will allow for better recognition and validation of patient experiences, ultimately improving outcomes in this disruptive condition.

This case series has several limitations, including self-diagnosis, subjective itch severity ratings, use of multi-ingredient supplements, potential placebo effects, and absence of objective measures. These factors limit causal inference and underscore that the observed effects are hypothesis-generating rather than confirmatory.

Beyond TRPV1-mediated desensitization, β-alanine may also engage other counter-stimulation pathways, including MRGPRD receptor activation [5], which could contribute to transient sensory neuron modulation and relief.

**Limitations:** This case series is based on self-reported symptoms without objective clinical or laboratory verification. Each patient self-diagnosed Hell’s Itch and used different β-alanine-containing pre-workout formulations with variable additional ingredients, creating potential confounding effects. Reported itch intensity scores were subjective and not standardized. Placebo effects or coincidental symptom resolution cannot be excluded. These findings are therefore anecdotal and intended to generate hypotheses for future controlled studies.

## 4. Conclusions

Hell’s Itch appears to involve non-histaminergic neuroimmune pathways; β-alanine may offer rapid symptomatic relief and warrants controlled evaluation [2]. The treatment of this condition focuses on symptomatic relief by targeting neuropathic itch pathways because antihistamines are ineffective in this population due to the non-histaminergic pathophysiology [8]. First-line therapies include topical anesthetics such as pramoxine, lidocaine, or menthol, which act on peripheral nerve fibers and are available over the counter [3]. For more severe or refractory cases, systemic treatments such as gabapentinoids, opioid receptor modulators (e.g., naltrexone), and certain antidepressants (e.g., sertraline and mirtazapine) have been used with varying success [3,9].

Future therapies are aimed at modulating the underlying immune and neuronal signaling, including Janus kinase (JAK) inhibitors, κ-opioid receptor agonists, biologics targeting IL-4, IL-13, and IL-31, and TRP channel antagonists [10].

This case report contributes to the evolving understanding of Hell’s Itch by describing the rapid symptomatic relief experienced with oral β-alanine, a supplement not traditionally used in dermatologic care. The patient’s improvement suggests a potential neuromodulatory effect, highlighting a novel treatment pathway that warrants further investigation. Increasing clinical recognition, research investment, and the inclusion of Hell’s Itch in dermatologic and primary care curricula may significantly improve patient outcomes and reduce the psychological burden of this condition.

## Figures and Tables

**Table 1:** Summary of case presentations.

Case	Age/Sex	Onset (Hours)	Severity (0–10)	Triggers	Treatments Tried	β-Alanine Dose	Time to Relief	Duration	Adverse Effects
1	26M	48–72	8–10	High altitude sun; outdoor activity	Antihistamines, acetaminophen, ibuprofen, aloe, lidocaine, cold therapy	3200–4800 mg (pre-workout)	5 min	2–3 h	Mild paresthesia
2	28M	48	9–10	Outdoor activity (golf, hiking)	Aloe, ibuprofen, lidocaine, ice, diphenhydramine, hot showers	1000 mg (RAW pre-workout)	30 min	2–3 h	None
3	23M	24	10	Boating outing, 95 °F (35 °C), sunburn + lukewarm shower	Ibuprofen, diphenhydramine, hot/cold showers, OTC creams	RYSE ~1600 mg (½ scoop; estimated from brand formulation).	5–10 min	3 h	Paresthesia-like tingling

β-alanine was taken in different pre-workout formulations containing other ingredients (e.g., caffeine, niacin, taurine, and betaine). Relief cannot be attributed solely to β-alanine.

## Data Availability

The data presented in this study are not publicly available due to privacy and ethical restrictions. De–identified case information is available from the corresponding author upon reasonable request and with approval from the Rocky Vista University Institutional Review Board.

## Abbreviations

The following abbreviations are used in this manuscript:

PHI	Protected Health Information
UVB	Ultraviolet B
TRPV1	Transient Receptor Potential Vanilloid 1
JAK	Janus Kinase
IL	Interleukin
UV	Ultraviolet
GPCR	G Protein-Coupled Receptor
